# Safety, Tolerability, and Efficacy of Pain Reduction by Gabapentin for Acute Headache and Meningismus After Aneurysmal Subarachnoid Hemorrhage: A Pilot Study

**DOI:** 10.3389/fneur.2020.00744

**Published:** 2020-07-28

**Authors:** Laxmi P. Dhakal, Marion T. Turnbull, Daniel A. Jackson, Emily Edwards, David O. Hodge, Neeharika Thottempudi, Prasuna Kamireddi, Oluwaseun O. Akinduro, David A. Miller, James F. Meschia, William D. Freeman

**Affiliations:** ^1^Department of Neurology, Mayo Clinic, Jacksonville, FL, United States; ^2^Department of Critical Care Medicine, Mayo Clinic, Jacksonville, FL, United States; ^3^Department of Pharmacy, Mayo Clinic, Jacksonville, FL, United States; ^4^Division of Biomedical Statistics and Informatics, Mayo Clinic, Jacksonville, FL, United States; ^5^Department of Neurologic Surgery, Mayo Clinic, Jacksonville, FL, United States; ^6^Division of Neuroradiology, Mayo Clinic, Jacksonville, FL, United States

**Keywords:** aneurysmal subarachnoid hemorrhage, gabapentin, headache, numeric pain scores, opioid

## Abstract

**Introduction:** Severe, often sudden-onset headache is the principal presenting symptoms of aneurysmal subarachnoid hemorrhage (aSAH). We hypothesized that gabapentin would be safe and tolerable for aSAH-induced headaches and would reduce concurrent opioid use.

**Methods:** We performed a single-center, double-blind, randomized, placebo-controlled trial (registered at ClinicalTrials.gov; NCT02330094) from November 24, 2014, to June 24, 2017, where aSAH patients received either dose-escalating gabapentin or oral placebo, both alongside a standard of care pain regimen. After 7 days, patients had the option to continue in an open-label period until 14 days after enrollment or until discharge from the intensive care unit. Our primary endpoint was the efficacy of gabapentin in reducing headache numeric pain scores and opioid usage in patients with aSAH compared to the placebo group. We identified 63 potential patients with aSAH for the study. After applying stringent exclusion criteria, 16 eligible patients were enrolled into one of two arms.

**Results:** The study ended prematurely after reaching a pre-specified funding period and an unexpected drop in aSAH cases. There was a trend toward lower headache numeric pain scores and opioid use in the gabapentin treated arm; however this was not significantly different. Gabapentin was well tolerated by participants and no adverse effects were reported.

**Conclusions:** While there was a trend toward lower pain scores and opioid requirement in the gabapentin group, the study was underpowered to detect a difference. Larger multicenter trials are required to evaluate the efficacy of gabapentin to reduce opioid requirements after aSAH.

## Introduction

Aneurysmal subarachnoid hemorrhage (aSAH) comprises ~6–8% of all strokes, with at least 30,000 new cases per year in Western countries (1). aSAH usually presents with a thunderclap headache, often described as the “worst headache imaginable,” and persists for days and weeks (2, 3). More recent studies demonstrate that a large proportion of aSAH patients continue to suffer from long term headaches which affects their healthcare-related quality of life (4).

Opioids are typically used for the management of aSAH-related pain in the hospital, and are frequently prescribed at discharge (5). Recently, the National Academies of Science and National Institutes of Health have raised awareness about the current public health crisis of misuse and addiction to opioids, and advise reducing opioid prescription when feasible (6). Opioids also have weighty adverse effects (AEs), including respiratory depression and arrest, obtundation, gastrointestinal ileus, constipation (20–60%), pruritus (0–30%), and urinary retention (40–45%) (7–11). High doses of opioids can also induce delirium, which may prolong intensive care unit (ICU) length of stay (10, 12). This has encouraged the investigation of other analgesic drugs for the management of severe headache experienced by aSAH patients in order to reduce opioid usage (13).

Gabapentin is an antiepileptic drug with well-known pain-modulating efficacy and is used successfully for neuropathic pain and perioperative pain indications (14), thus we hypothesized that gabapentin would be safe and tolerable for patients with aSAH and could result in reduced opioid use. We have previously reported on the safety and tolerability of gabapentin use in a single-center retrospective study of 53 patients with aSAH (15). Gabapentin was used alongside other analgesics, with nausea in 6% of cases (intolerable in 1.8%), but no major AEs (15)

We conducted a randomized, double-blind, placebo-controlled study to investigate safety and tolerability of gabapentin use for aSAH-related headache. Moreover, we aimed to assess the efficacy of gabapentin for headache treatment in patients with aSAH and its potential for reducing opioid use.

## Methods

### Study Overview

This study was designed as part of a research scientific thesis (L. P. D.) for the Mayo Clinic Center for Clinical and Translational Science research program. It was reviewed for scientific merit by the Mayo Clinic Neuroscience Focused Research Team, received Mayo Clinic Institutional Review Board (IRB) approval, and was registered on ClinicalTrials.gov (NCT02330094). The study was internally funded and was determined by the FDA and Mayo Clinic IRB to be IND-exempt, since our use of gabapentin fell within the drug's primary indication for pain reduction (neuropathic and non-neuropathic) and not with the intent of providing evidence for a change in label indication. We also provided evidence of safety and tolerability from our prior retrospective study to the FDA (15).

### Inclusion and Exclusion Criteria

Patients were eligible to participate if they were 18 years of age or older, had an aSAH, were able to swallow pills and verbalize pain score, and had a numeric pain score (NPS) of at least 5 prior to randomization. Informed consent was obtained from all patients. Exclusion criteria included gabapentin use prior to hospital admission for aSAH, creatinine clearance (CrCl) <30 mL/min, pregnancy, history of severe depression defined by DSM IV criteria, or incapacitation or inability to provide consent.

### Study Protocol and Pharmacy Double Blind Methods

After IRB approval, we evaluated aSAH cases admitted to the Neuroscience ICU (NeuroICU) at Mayo Clinic in Jacksonville, Florida, from November 24, 2014, to June 24, 2017. Enrollment was performed within 72 h of hospital admission and after meeting inclusion criteria.

The Mayo Clinic Department of Pharmacy implemented allocation for the double-blind drug or placebo concealment and used permuted block randomization to ensure an equal number of patients in each group as the study progressed. Gabapentin and placebo medications, provided by the central pharmacy, were compounded and repackaged to look identical.

### Pain Assessment and Management

A bedside nurse recorded the NPS (0–10, 0 being no pain and 10 being worst pain) every 4 h per hospital policy. The total score over 24 h was averaged as the daily NPS. Only headache and neck pain (meningismus) were included in the study to calculate the pain score. After consent and enrollment, nursing staff recorded data collection sheets with daily NPS scores and standard of care (SOC) management plans. SOC pain management plans were implemented by the NeuroICU team of residents, attendings, nurse practitioners, and physician assistants. A decrease in NPS of >50% from the previous day's NPS was defined as good pain control and would not require escalation of gabapentin or placebo for that day.

### Patient Laboratory and Vasospasm Monitoring

We reviewed CrCl, blood urea nitrogen, and liver function tests from the medical record performed as SOC by the treating team. The team reviewed patient computed tomography, computed tomography angiography, transcranial Doppler, and vasospasm interventions (e.g., cerebral angiography). aSAH severity was assessed at baseline using the modified Fisher scale.

### Gabapentin Dose Escalation and Monitoring

The gabapentin group was given active medication with possible escalation according to CrCl (Supplementary Table 1). Gabapentin escalation dosing was modeled after our retrospective safety study (15). We started with 100 mg orally three times daily, titrating up every 24 h to a maximum of 900 mg three times daily until we reached a decrease of 50% or more on average NPS. The gabapentin maintenance dose was defined as the dosage given for 24 h after which there was 50% reduction of daily average NPS. The placebo group was escalated in the same manner as the gabapentin group. Patients opting to continue gabapentin beyond the study were followed by local neurologists to safely reduce or stop gabapentin. The National Cancer Institute Common Terminology Criteria for Adverse Events was used for reporting adverse events and grading severity. Each patient's total daily dose of gabapentin was tabulated for every hospital day. Laboratory values were reviewed for abnormality in hepatic or renal function.

After the 7-day study period, patients were unblinded and could crossover to receive gabapentin if their head and neck pain was still not well controlled with the SOC pain management regimen alone (Supplementary Figure 2). Gabapentin dose escalation during the open-label phase followed the same escalation guidelines as the main study period.

### Meningitis Screen

Whenever CSF was collected by SOC via an external ventricular drain or lumbar puncture, we measured white (WBC) and red blood cell (RBC) counts to calculate the CSF WBC/RBC ratio, and culture and sensitivity for evidence of bacterial meningitis.

### Opioid Requirement

We calculated total morphine equivalent (ME) dose every day with an opiate conversion table (Supplementary Table 2) (16).

### Patient Pain Satisfaction Score

We used the modified Brigham and Women's Hospital Management of Postoperative Pain Patients discharge questionnaire (BWQ) (Supplementary Table 3), which has an average test-retest reliability of r equals 0.86, with a range of coefficients from 0.76 to 0.92, and interexaminer reliability of r equals 0.98 (17). The score was calculated at day 8 after completion of the primary endpoint of our study by study investigators (Drs. Dhakal, Freeman, and Ms. Edwards).

### Sample Size

Our previous retrospective study demonstrated a 25% difference in NPS daily score between gabapentin and placebo groups and a mean (SD) daily pain score of 26 (5.84) at day 4 (15). For our current study, we expected the mean percentage difference in NPS score would be 25%. Assuming α was kept at 0.05 and power at 80%, the sample size predicted would be <10 patients per group to reject the null hypothesis, with an overall goal of 20 patients.

### Statistical Methods

The primary endpoint for this trial was 7 days of treatment with or without gabapentin and SOC, and the treatment groups were compared using a 2-tailed test with a *P*-value of 0.05.

**Figure 1:**
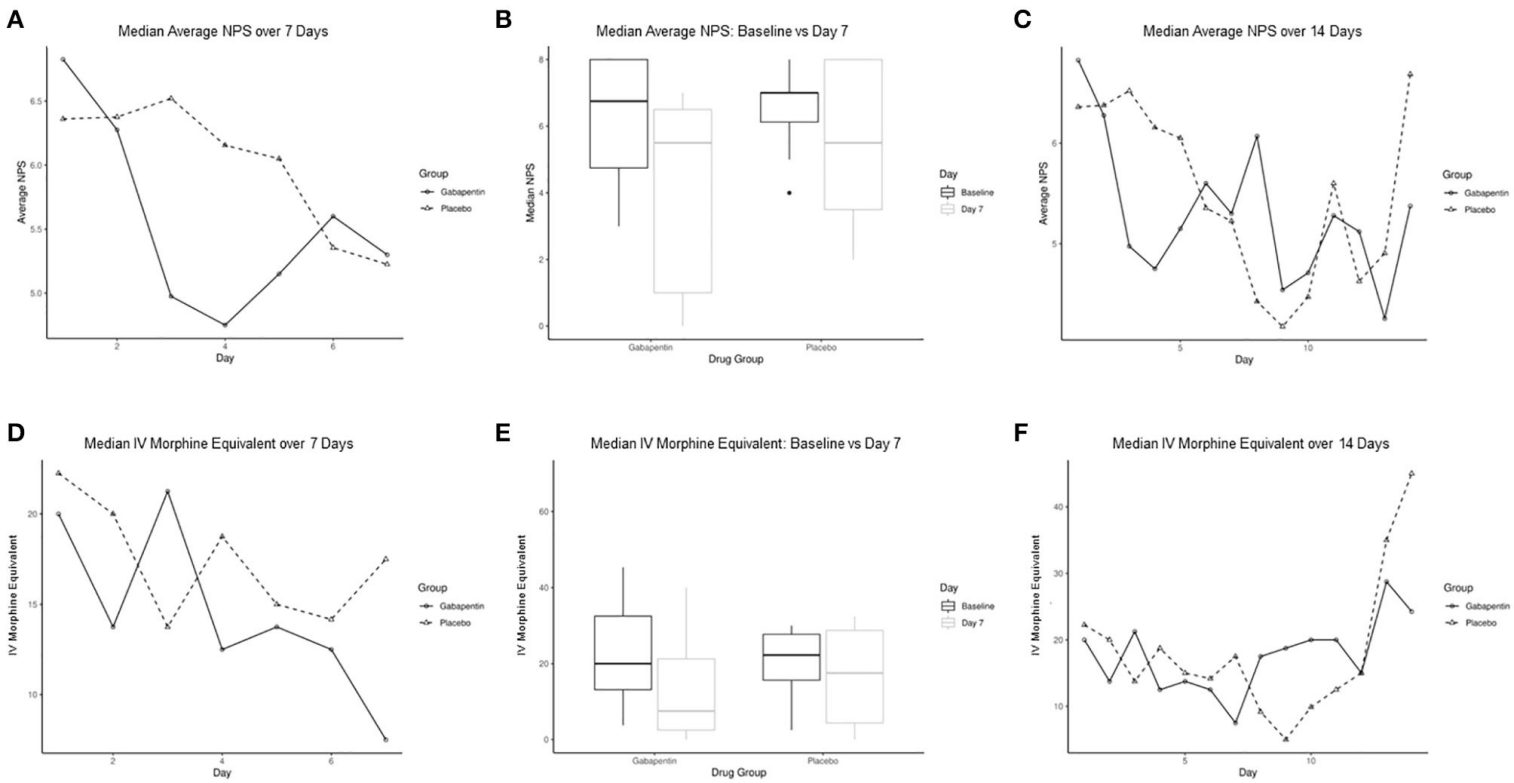
Comparison of median average numeric pain score (NPS) and IV morphine equivalent per day between gabapentin and placebo groups. **(A)** Median average NPS between groups for the first 7 days. **(B)** Box graphs of median average NPS at baseline compared to day 7 for gabapentin and placebo groups. **(C)** Median average NPS between groups over 14 days. **(D)** Median IV morphine equivalent between groups for first 7 days. **(E)** Box graphs of median IV morphine equivalent at baseline compared to day 7 for gabapentin and placebo groups. **(F)** Median IV morphine equivalent between groups over 14 days. IV indicates intravenous; NPS, numeric pain score.

**Figure 2:**
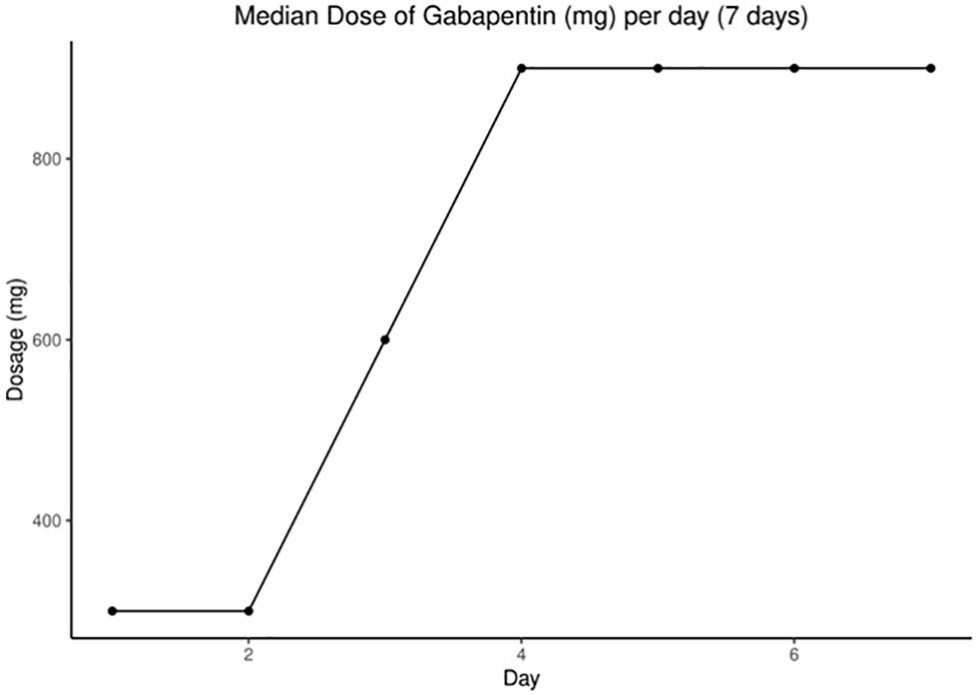
Increasing median gabapentin dose over first 7 days.

Categorical variables were summarized as percentages, and continuous factors were described using means or medians to best describe the distributions. AEs were described as rates. Comparisons in categorical variables between groups were completed using the χ^2^ test for independence. Daily average NPS score, opioid requirement, and gabapentin dose were plotted graphically (Figures 1, 2). Continuous variables were compared using the Wilcoxon rank-sum test.

Overall comparisons of NPS, patient satisfaction score, and the fentanyl requirement were reviewed over the first 7 days, using a repeated measures analysis of variance. Furthermore, values were averaged over those days, and individual measures were compared at 7 and 14 days. Those comparisons were made between groups using Wilcoxon rank-sum tests. SAS (version 9.4) was used for statistical analysis.

## Results

From November 24, 2014, to June 24, 2017, we screened 63 patients with aSAH, of whom 16 met final eligibility criteria and were enrolled into the trial. Our study was stopped prematurely due to a decline in recruitment and a pre-specified time period being met.

### Demographics

Sixteen patients were randomized evenly into gabapentin (*n* = 8) and placebo (*n* = 8) groups. Median (range) age was 51 (37.5, 71.8) years and 12 (75%) were women: four (50.0%) in the gabapentin group and all eight (100.0%) in the placebo group (*P* = 0.02). Overall, there was no significant difference in median (range) age between groups [53.62 (38.1–69.7), 45.57 (37.5–71.8), *P* = 0.53]; however, the gabapentin group had higher aSAH severity scores (*P* = 0.01) as measured by modified Fisher Scale. All patients enrolled in the trial had imaging studies to confirm an aSAH diagnosis. The most common type of aneurysm noted was middle cerebral artery (*n* = 5), followed by anterior communicating artery (*n* = 4). Four patients in the gabapentin group were reported with baseline chronic pain symptoms, and one was on chronic pain medication. Baseline demographic factors compared between the groups are provided in Table 1.

**Table 1:** Patient demographics and characteristics.

**Characteristics**	**Gabapentin (*n* = 8)**	**Placebo (*n* = 8)**	**Total (*n* = 16)**	***P*-Value**
Age, y				0.53
Mean (SD)	52.7 (10.2)	49.8 (11.6)	51.3 (10.6)	
Median (range)	53.62 (38.1–69.7)	45.57 (37.5–71.8)	50.56 (37.5–71.8)	
Sex, No. (%)				0.02
Female	4 (50.0)	8 (100.0)	12 (75.0)	
Racial category, No. (%)				0.55
Black	2 (25.0)	3 (37.5)	5 (31.3)	
White	5 (62.5)	5 (62.5)	10 (62.5)	
Unknown or not reported	1 (12.5)	0 (0.0)	1 (6.3)	
Modified Fisher grade, No. (%)				0.01
1	0 (0.0)	0	0	
2	0 (0.0)	2 (25.0)	2 (12.5)	
3	0 (0.0)	4 (50.0)	4 (25.0)	
4	8 (100.0)	2 (25.0)	10 (62.5)	
Chronic pain, No. (%)				0.61
Yes	4 (50.0)	5 (62.5)	9 (56.3)	
Pain medication use, No. (%)				0.25
Yes	1 (12.5)	3 (37.5)	4 (25.0)	

Two patients in the gabapentin group withdrew from the study, one at day 5 and the other at day 7. Both patients had worsening vasospasm related to their clinical diagnosis of aSAH, deemed by the physician investigator to not be related to gabapentin or opioid use. These patients were intubated, and thus were unable to report NPS. Overall, five patients in the placebo group were started with gabapentin escalation during the open label timeframe. Three episodes of vasospasm were detected by transcranial Doppler throughout the study; two were in the gabapentin group and one in the placebo group. The average time to enrollment was 6.2 days after aSAH onset.

### NPS and Opioid Use

The median (range) average NPS for the gabapentin and placebo groups from enrollment through day 7 were 5.4 (2.4, 7.7) and 5.7 (3.5, 7.7), respectively (*P* = 0.70). Similarly, there was a nonsignificant trend in median NPS, intravenous fentanyl use, and MEs. When comparing NPS at day 7, there was a trend for the gabapentin group to have lower pain scores than the placebo group [5.3 (0.0, 6.4) vs. 5.2 (1.4, 7.3), *P* = 0.27], which continued at day 14 [5.4 (0.0, 7.3) vs. 6.7 (4.6, 8.8), *P* = 0.81] (Figures 1A–C). However, this was not significant, likely due to inadequate sample size. ME requirement also showed initial higher scores in the gabapentin group compared to placebo at day 7 [7.5 (0.0, 40.0) vs. 17.5 (0.0, 70.0), *P* = 0.60] (Figures 1D–F).

### Gabapentin

The median (range) dose of gabapentin at the end of day 7 was 900 (400, 2,700) mg (Figure 2). Rapid escalation of gabapentin was achieved in most patients without AEs, assuming normal CrCl. Table 2 demonstrates nonsignificant differences in gabapentin compared to placebo in terms of median or mean NPS and MEs by day 7. We performed a repeated measures analysis of variance, as we had measured data from the same patients over time, focusing on the differences between the gabapentin and placebo groups. After adjusting for time, there was no significant difference between groups for the mean (*P* = 0.40) or median NPS scores (*P* = 0.38). The same analysis was performed for the other variables, but the results were not statistically significant for particular opioids used (e.g., fentanyl use, *P* = 0.99; ME, *P* = 0.92).

**Table 2:** Gabapentin and placebo comparison during the first 7 days of study.

**Numeric pain scores (NPS) and dosing**	**Gabapentin (*n* = 8)**	**Placebo (*n* = 8)**	**Total (*n* = 16)**	***P*-Value**
Median NPS				0.60
Mean (SD)	5.3 (2.0)	6.0 (1.6)	5.7 (1.8)	
Median (range)	5.6 (2.4–8.1)	5.6 (3.6–8.1)	5.6 (2.4–8.1)	
Average NPS				0.79
Mean (SD)	5.2 (1.9)	5.8 (1.5)	5.5 (1.7)	
Median (range)	5.4 (2.4–7.7)	5.7 (3.5–7.7)	5.4 (2.4–7.7)	
Gabapentin Dose (mg)				–
Mean (SD)	961.1 (603.8)	–	961.1 (603.8)	
Median (range)	678.6 (400.0–1,971.4)	–	678.6 (400.0–1,971.4)	
Morphine equivalent				0.79
Mean (SD)	19.4 (14.8)	20.2 (13.1)	19.8 (13.5)	
Median (range)	13.3 (5.4–45.5)	17.1 (5.0–44.5)	14.3 (5.0–45.5)	

–*, not applicable; NPS, numeric pain scores*.

### Patient Satisfaction Score

For statistical calculation, we transformed the BWQ score (Supplementary Table 3) into a uniform scale, where all numbers and sections went the same direction, so that a lower score would indicate a better outcome. The total median (range) BWQ score for the gabapentin group at the end of Day 7 was not significantly different to the placebo group [13.5 (8.0, 20.0) vs. 14.0 (8.0, 16.0), *P* = 0.66], although there was a trend toward a better satisfaction score.

### Safety and Adverse Events Reporting

There was one episode of nausea and agitation recorded in each group. In the placebo group, there was also one episode of delirium and one of vomiting (Supplementary Table 4). There were no significant differences between the groups when comparing AEs. Blood urea nitrogen ranged between 3 and 24 mg/dL (normal, 7–20) and CrCl ranged between 0.3 and 1.5 mg/dL (normal, 0.6–1.0). We did not observe unexplained worsening of liver or kidney function. Overall, we noted no major AEs.

### CSF WBC/RBC Ratio, Aseptic Meningitis, and CSF Culture Results

There is little known regarding the CSF WBC/RBC ratio in aSAH patients aside from our prior retrospective gabapentin study (15). While it is known the corticosteroids can cause peripheral WBC count elevation on blood tests, we are uncertain what effect dexamethasone would have on CSF WBC/RBC ratios. Nonetheless, we report that four patients had high (>10 mg) dexamethasone use in the first 2 days of the study, but use later dropped off. CSF cultures for all patients were negative for microorganisms throughout the study.

## Discussion

**Figure 3:**
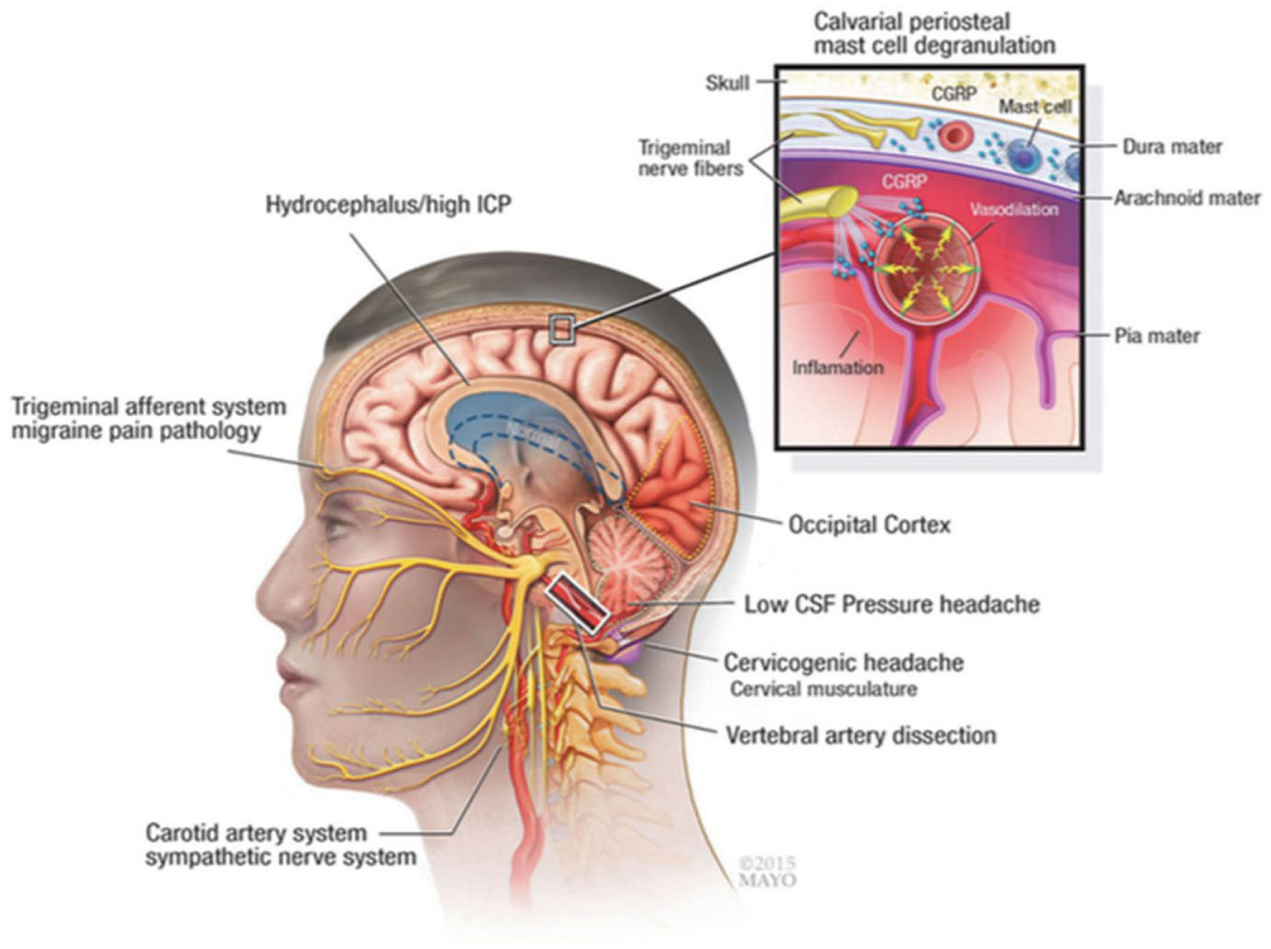
Pathophysiologic mechanisms of headache and meningeal irritation after subarachnoid hemorrhage and other common causes, such as migraine and cervicogenic headache. CGRP indicates calcitonin gene-related peptide; CSF, cerebrospinal fluid; ICP, intracranial pressure. Used with permission of Mayo Foundation for Medical Education and Research. All rights reserved.

The current study investigated the use of gabapentin vs. placebo for headache pain relief in patients with aSAH while admitted to a NeuroICU as gabapentin can be used safely for days to weeks for pain management. This is the first randomized trial to date reporting the use of gabapentin to reduce headache pain in these patients. Headache pathophysiology after aSAH is complex and involves influences from raised intracranial pressure and potential hydrocephalus initially to delayed, sterile neuroinflammatory response from blood products irritating the meninges (Figure 3). Numerous putative neuropathic and trigeminovascular pathways are involved with aSAH-related headache.

Gabapentin is commonly used for its anti-nociceptive effects, especially in anesthesia during the perioperative period, and has been shown to reduce analgesic, particularly opiate, consumption and associated AEs (18). The 2013 Clinical Practice Guidelines for the Management of Pain, Agitation, and Delirium in Adult Patients in the Intensive Care Unit has also recommended enteral administration of gabapentin, in addition to intravenous opioids, for treatment of neuropathic pain (+1A) (11). Patients may also benefit from gabapentin's anxiolytic, antiemetic, antipruritic, and anti-inflammatory effects (19, 20). Additionally, given its current use as an anticonvulsant, gabapentin would be ideal for patients with aSAH at risk for seizures (21). Therefore, we believe gabapentin is particularly suited for pain management of headache for patients with aSAH.

Although the exact molecular mechanisms of action by which gabapentin mediates its effects are not yet fully understood, current evidence suggests that it binds to and modulates the α2 δ-1 subunit of voltage-gated calcium channels {(22) #21; (23) #1055; (24) #8}. Moreover, it may mediate anti-neuroinflammatory effects by preventing substance P-induced cytokine production and calcitonin gene-related peptide (CGRP) release (25). This is particularly relevant to patients with aSAH as there is a large inflammatory component to the disease. We have shown that the secondary wave of aSAH-induced headaches correlate with an aseptic inflammation of the meninges, possibly caused by the breakdown of blood products, which aggravates head, neck and back pain (15). Thus, gabapentin's anti-inflammatory properties could potentially contribute to better patient outcomes.

Our trial has several strengths, including the randomized, double-blind, placebo-controlled design and the 7-day single crossover. The initial 7-day period was designed to assess the primary endpoint, which was pain reduction and reduced opioid use, while still maintaining effective pain management. Although there was a trend toward opioid reduction, the study was ultimately underpowered and stopped after an interim analysis and pre-specified funding period ended, which showed non-inferiority of safety, tolerability, and pain endpoints. A confounding factor in our study was that the gabapentin group started with higher aSAH severity scores than the placebo group. Moreover, there were more women in the placebo control group - although this reflects the gender distribution of aSAH cases and will need to be addressed in future studies.

This data can be used as a pilot study to design a larger prospective and multicenter clinical trial. Inclusion and exclusion criteria were stringent, excluding many patients with aSAH; however, a homogenous population was required. Another limitation to our study is the subjective nature of NPSs. While vital signs and other measured variables may suggest more physiologic pain, NPSs can be influenced by factors such as anxiety, depression, and baseline pain tolerance, which affect reporting by patients. Furthermore, we had to exclude comatose and nonverbal patients with aSAH as they would have been unable to respond, which contributed to lower enrollment volumes. Nonetheless, the study was designed in a randomized fashion to help account for these covariates in both groups.

Our study investigated use of gabapentin vs. placebo for headache in patients with aSAH able to verbalize their NPS while admitted to a neuroICU. This study confirms our previous retrospective study demonstrating that gabapentin is safe and tolerable in patients with aSAH (15), however we were unable to demonstrate efficacy due to our small sample size. To our knowledge, this is the first randomized trial of gabapentin use for aSAH-associated pain control. In the future, larger numbers of patients will be needed to demonstrate the efficacy of gabapentin and whether it can reduce concurrent opioid use.

## Conclusions

This is the first preliminary randomized controlled trial of gabapentin vs. placebo for headache and meningismus in patients with aSAH in a NeuroICU environment. While the study was underpowered for efficacy, there was a trend toward lower concurrent opioid use and lower overall NPS reporting in the gabapentin group. Gabapentin appears safe and tolerable for patients with aSAH to manage major headache pain and meningismus.

## Data Availability Statement

The datasets generated for this study are available on request to the corresponding author.

## Ethics Statement

The studies involving human participants were reviewed and approved by Mayo Clinic Institutional Review Board. The patients/participants provided their written informed consent to participate in this study.

## Author Contributions

LD contributed to conception and design, experiments, collection, analysis and interpretation of data, drafting and critical revision of the manuscript, and collection/generation of tables and figures. MT contributed to analysis and interpretation of data and critical revision of the manuscript. DJ contributed to experiments, collection of data, and critical revision of the manuscript. EE contributed to conception and design, experiments, collection, analysis and interpretation of data, and drafting and critical revision of the manuscript. DH contributed to collection, analysis and interpretation of data, and critical revision of the manuscript. NT, PK, and OA contributed to collection of data and critical revision of the manuscript. DM and JM contributed to conception and design, experiments, and critical revision of the manuscript. WF contributed to conception and design, experiments, collection, analysis and interpretation of data, drafting and critical revision of the manuscript, and collection/generation of tables and figures. All authors contributed to the article and approved the submitted version.

## Conflict of Interest

The authors declare that the research was conducted in the absence of any commercial or financial relationships that could be construed as a potential conflict of interest.
