# Supplementary figures and images for: Safety, Tolerability, and Efficacy of Pain Reduction by Gabapentin for Acute Headache and Meningismus After Aneurysmal Subarachnoid Hemorrhage: A Pilot Study
Source: Front Neurol. 2020 Jul 28;11:744. doi: 10.3389/fneur.2020.00744 (PMC7399216; doi:10.3389/fneur.2020.00744)

## CONSORT 2010 Flow Diagram

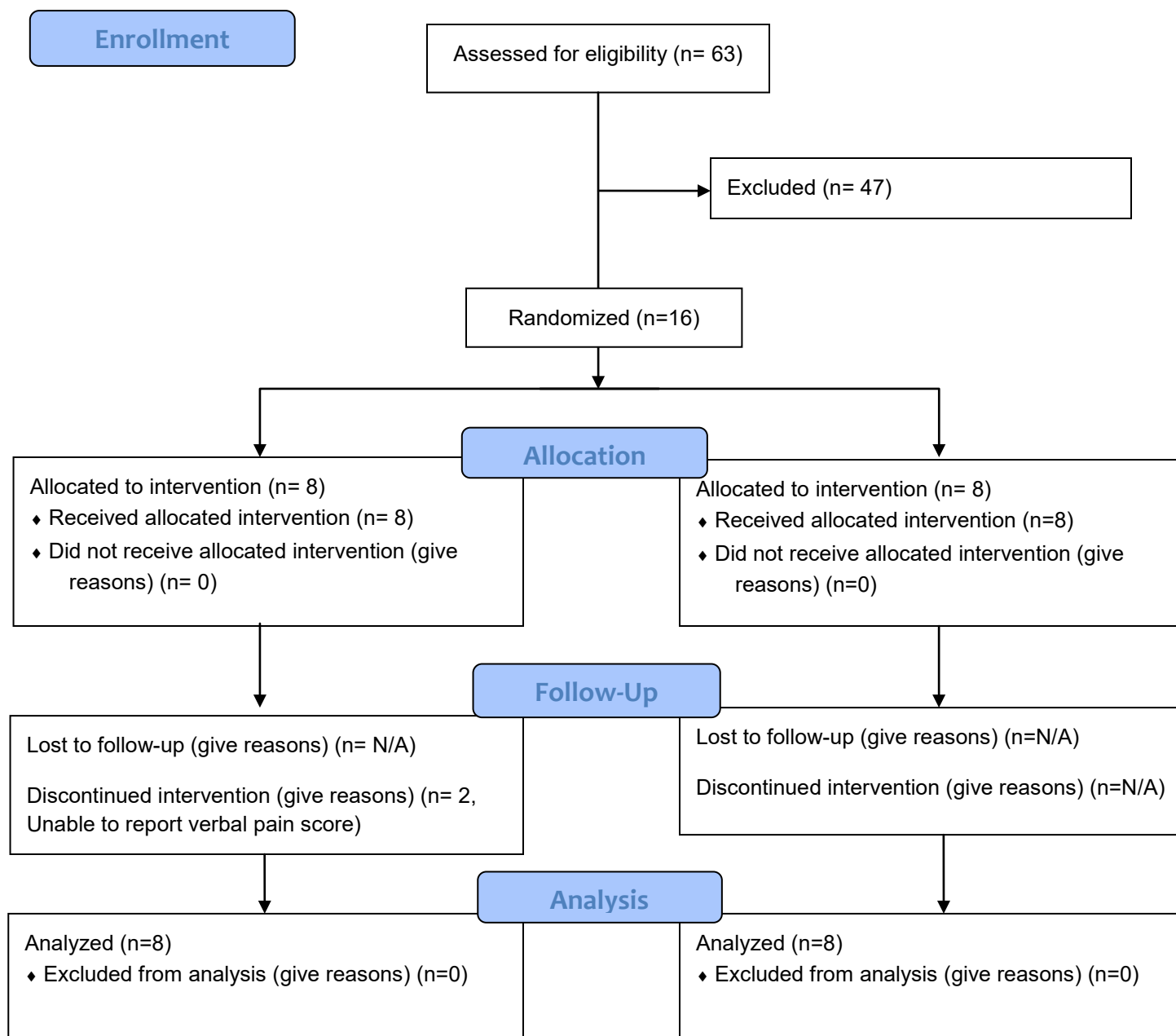

Supplement: Supplementary file 1 [file Data_Sheet_1.PDF]

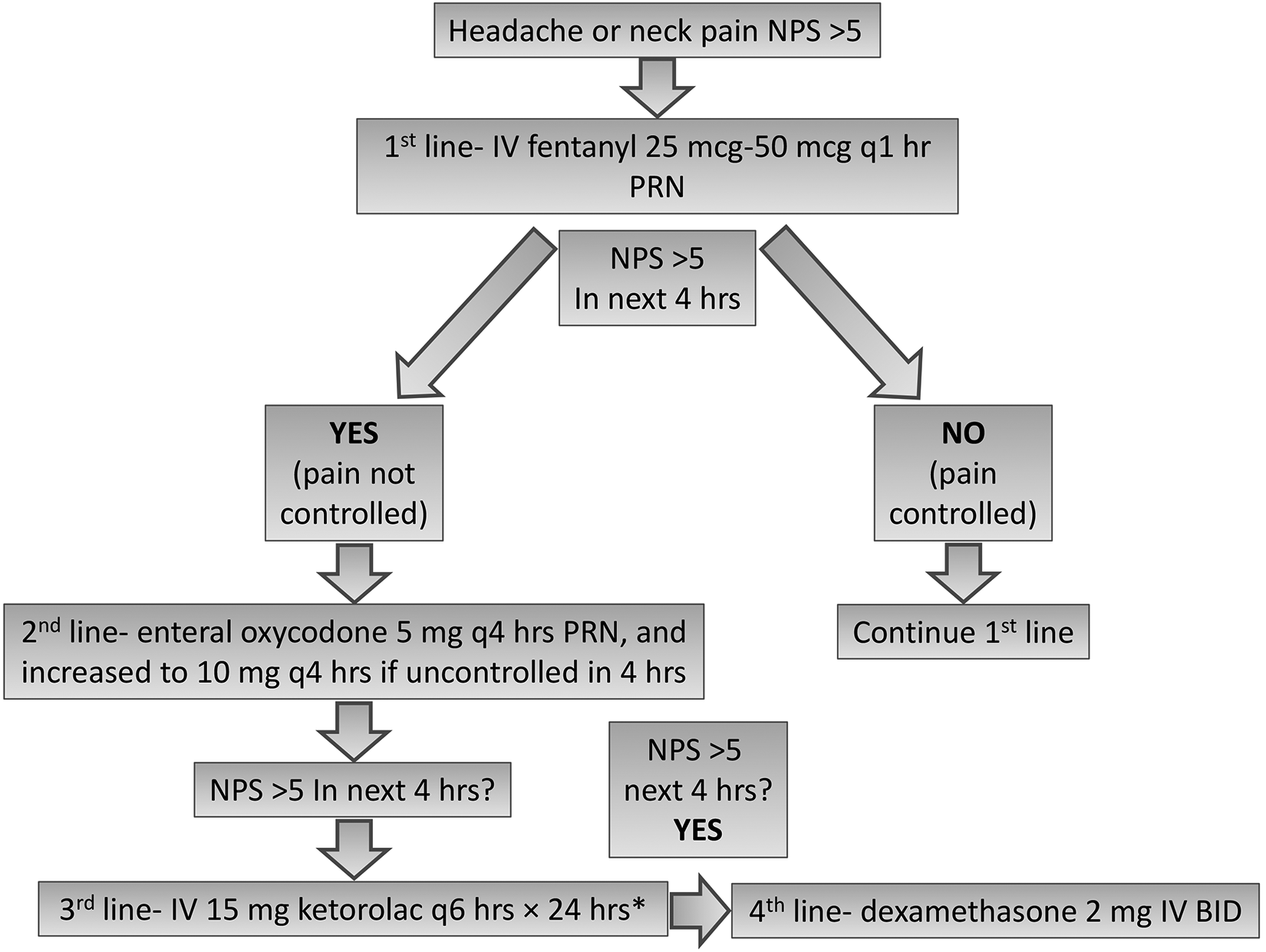

Supplement: Supplementary file 3 [file Image_1.TIF]

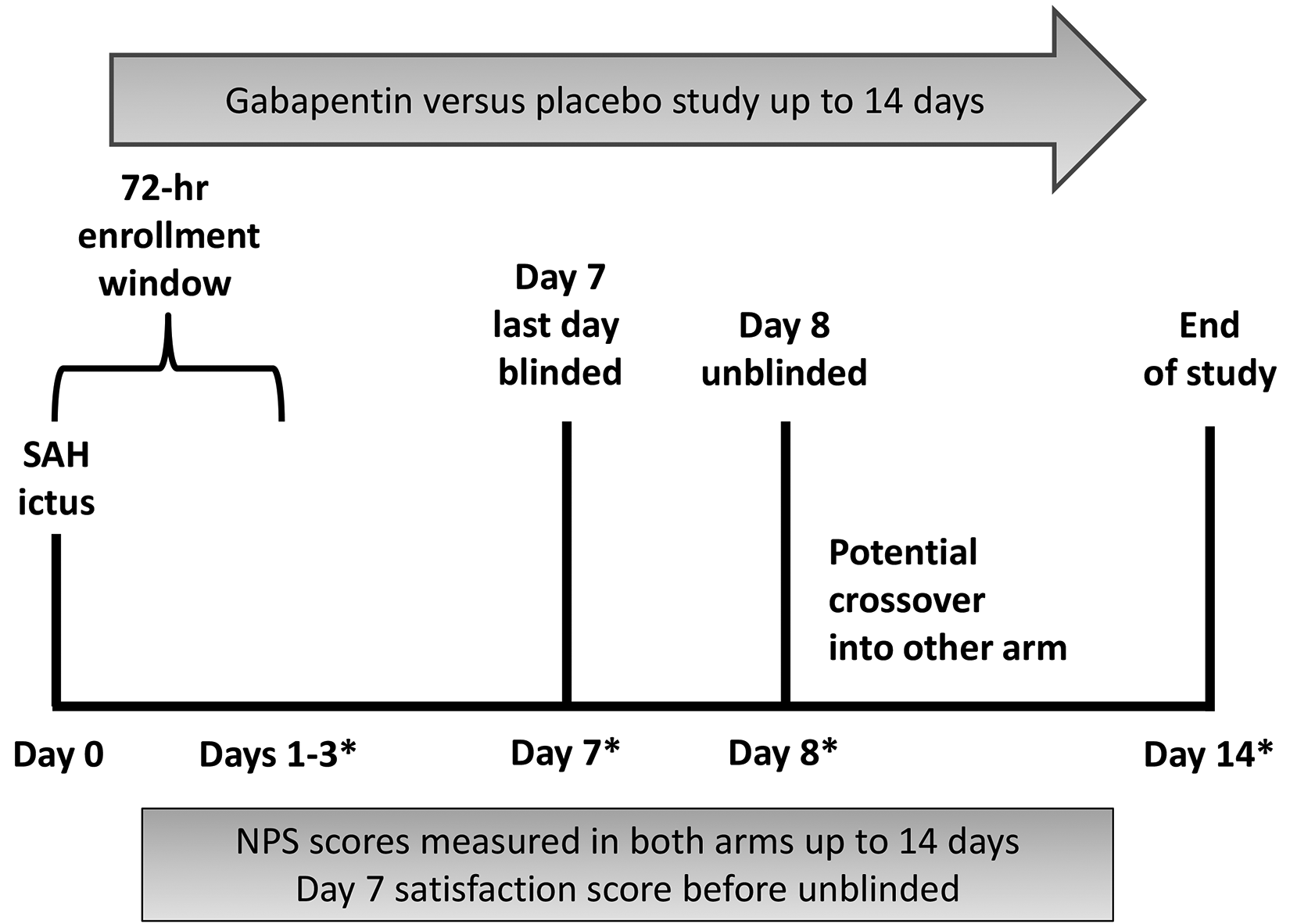

Supplement: Supplementary file 4 [file Image_2.TIF]
