# Supplementary material for: Safety, Tolerability, and Efficacy of Pain Reduction by Gabapentin for Acute Headache and Meningismus After Aneurysmal Subarachnoid Hemorrhage: A Pilot Study
Source: Front Neurol. 2020 Jul 28;11:744. doi: 10.3389/fneur.2020.00744 (PMC7399216; doi:10.3389/fneur.2020.00744)
Supplement: Supplementary file 2 [file Data_Sheet_2.docx]

Supplementary Material

**Supplemental Tables**

**Supplemental Table 1.** Gabapentin Dosage Based on Renal Function

| Renal Function Creatinine Clearance (mL/min) | Maximum Total Daily Dose (mg/day) |
| --- | --- |
| ≥60 | 2,700 |
| >30–59 | 1,200 |

**Supplemental Table 2.** Morphine Equivalence Opiate Conversion^a^

| Product | Equianalgesic Oral Morphine | Equianalgesic Parenteral (IV) Morphine |
| --- | --- | --- |
| Morphine | 2 mg | 1 mg |
| Fentanyl (IV) 25 mcg | -- | 2.5 mg |
| Hydromorphone (IV) 1 mg | -- | 7.5 mg |
| Oxycodone (PO) 5 mg | 7.5-10 mg | 2.5 mg |

Abbreviations: --, not applicable; IV, intravenous; PO, per oral.

^a^Adapted from Shaheen PE, Walsh D, Lasheen W, Davis MP, Lagman RL. Opioid equianalgesic tables: Are they all equally dangerous? J Pain Symptom Manage 2009;38:409-417, under the terms of the Creative Commons Attribution-NonCommercial-No Derivatives License (CC BY NC ND).

**Supplemental Table 3.** Modified Brigham and Women’s Hospital Pain Management Satisfaction Score^a^

| **A. Questions** | **0**  **No pain** | **1**  **Mild pain** | **2**  **Moderate** | **3**  **Severe** | **4**  **Very Severe** | **5**  **Unbearable** | **Comments** |
| --- | --- | --- | --- | --- | --- | --- | --- |
| 1. How much pain do you have right now? |  |  |  |  |  |  |  |
| 2. How much pain have you had in past 24 hrs? |  |  |  |  |  |  |  |
| **B. Questions** | **1**  **Strongly agree** | **2**  **Agree** | **3**  **Neutral** | **4**  **Disagree** | **5**  **Strongly disagree** | **Comments** | |
| 1. I am satisfied with the way the nurse and physician treated my pain. |  |  |  |  |  |  |  |
| 2. I am dissatisfied with the care I received during my hospitalization. |  |  |  |  |  |  |  |
| 3. The physician and nurse were concerned with how much pain I might be experiencing. |  |  |  |  |  |  |  |
| **C. Questions** | **0**  **Not at all** | **1**  **Barely** | **2**  **Slightly** | **3**  **Moderately** | **4**  **Quite a bit** | **5**  **Extremely** | |
| 1. How helpful were the medication and other treatments in relieving your pain? |  |  |  |  |  |  | |

^a^Used with permission: Jamison RN, Ross MJ, Hoopman P, Griffin F, Levy J, Daly M, et al. Assessment of postoperative pain management: patient satisfaction and perceived helpfulness. Clin J Pain. 1997 Sep;13(3):229-36.

**Supplemental Table 4.** Adverse event tracking by group

|  | **Gabapentin (n=8)** | **Placebo (n=8)** | **Total (n=17)** | ***P* value** |
| --- | --- | --- | --- | --- |
| **Miosis** |  |  |  |  |
| No | 8 (100.0%) | 8 (100.0%) | 16 (100.0%) |  |
|  |  |  |  |  |
| **Opioid constipation** |  |  |  |  |
| No | 8 (100.0%) | 8 (100.0%) | 16 (100.0%) |  |
|  |  |  |  |  |
| **CNS** |  |  |  |  |
| No | 8 (100.0%) | 8 (100.0%) | 16 (100.0%) |  |
|  |  |  |  |  |
| **Dry mouth** |  |  |  |  |
| No | 8 (100.0%) | 8 (100.0%) | 16 (100.0%) |  |
|  |  |  |  |  |
| **Myoclonus** |  |  |  |  |
| No | 8 (100.0%) | 8 (100.0%) | 16 (100.0%) |  |
|  |  |  |  |  |
| **Nausea** |  |  |  | 1.00 |
| No | 7 (87.5%) | 7 (87.5%) | 14 (87.5%) |  |
| Yes | 1 (12.5%) | 1 (12.5%) | 2 (12.5%) |  |
|  |  |  |  |  |
| **Delirium** |  |  |  | 1.00 |
| No | 8 (100.0%) | 7 (87.5%) | 15 (93.8%) |  |
| Yes | 0 (0.0%) | 1 (12.5%) | 1 (6.3%) |  |
|  |  |  |  |  |
| **Vomit** |  |  |  | 1.00 |
| No | 8 (100.0%) | 7 (87.5%) | 15 (93.8%) |  |
| Yes | 0 (0.0%) | 1 (12.5%) | 1 (6.3%) |  |
|  |  |  |  |  |
| **Pruritis** |  |  |  |  |
| No | 8 (100.0%) | 8 (100.0%) | 16 (100.0%) |  |
|  |  |  |  |  |
| **Rigidity** |  |  |  |  |
| No | 8 (100.0%) | 8 (100.0%) | 16 (100.0%) |  |
|  |  |  |  |  |
| **Serotonin** |  |  |  |  |
| No | 8 (100.0%) | 8 (100.0%) | 16 (100.0%) |  |
|  |  |  |  |  |
| **Urinary** |  |  |  |  |
| No | 8 (100.0%) | 8 (100.0%) | 16 (100.0%) |  |
|  |  |  |  |  |
| **Withdrawal** |  |  |  |  |
| No | 8 (100.0%) | 8 (100.0%) | 16 (100.0%) |  |
|  |  |  |  |  |
| **Other** |  |  |  | 1.00 |
| No | 7 (87.5%) | 8 (100.0%) | 15 (93.8%) |  |
| Yes | 1 (12.5%) | 0 (0.0%) | 1 (6.3%) |  |
|  |  |  |  |  |
| **Acute bacterial** |  |  |  |  |
| No | 8 (100.0%) | 8 (100.0%) | 16 (100.0%) |  |
|  |  |  |  |  |
| **Agitation** |  |  |  | 1.00 |
| No | 7 (87.5%) | 7 (87.5%) | 14 (87.5%) |  |
| Yes | 1 (12.5%) | 1 (12.5%) | 2 (12.5%) |  |
|  |  |  |  |  |
| **Atrial Fibrillation** |  |  |  | 1.00 |
| No | 7 (87.5%) | 8 (100.0%) | 15 (93.8%) |  |
| Yes | 1 (12.5%) | 0 (0.0%) | 1 (6.3%) |  |
|  |  |  |  |  |
| **Blood clots in urine** |  |  |  | 1.00 |
| No | 7 (87.5%) | 8 (100.0%) | 15 (93.8%) |  |
| Yes | 1 (12.5%) | 0 (0.0%) | 1 (6.3%) |  |
|  |  |  |  |  |
| **Cerebral Salt Wasting** |  |  |  | 1.00 |
| No | 8 (100.0%) | 7 (87.5%) | 15 (93.8%) |  |
| Yes | 0 (0.0%) | 1 (12.5%) | 1 (6.3%) |  |
|  |  |  |  |  |
| **CSF Leak** |  |  |  | 1.00 |
| No | 7 (87.5%) | 8 (100.0%) | 15 (93.8%) |  |
| Yes | 1 (12.5%) | 0 (0.0%) | 1 (6.3%) |  |
|  |  |  |  |  |
| **Diarrhea** |  |  |  | 1.00 |
| No | 7 (87.5%) | 8 (100.0%) | 15 (93.8%) |  |
| Yes | 1 (12.5%) | 0 (0.0%) | 1 (6.3%) |  |
|  |  |  |  |  |
| **Emesis** |  |  |  | 1.00 |
| No | 7 (87.5%) | 8 (100.0%) | 15 (93.8%) |  |
| Yes | 1 (12.5%) | 0 (0.0%) | 1 (6.3%) |  |
|  |  |  |  |  |
| **Fever** |  |  |  | 0.57 |
| No | 5 (62.5%) | 7 (87.5%) | 12 (75.0%) |  |
| Yes | 3 (37.5%) | 1 (12.5%) | 4 (25.0%) |  |
|  |  |  |  |  |
| **Hyperammonemia** |  |  |  |  |
| Missing | 7 | 8 | 15 |  |
| Yes | 1 (100.0%) | 0 (0.0%) | 1 (100.0%) |  |
|  |  |  |  |  |
| **Hypernatremia** |  |  |  | 0.47 |
| No | 8 (100.0%) | 6 (75.0%) | 14 (87.5%) |  |
| Yes | 0 (0.0%) | 2 (25.0%) | 2 (12.5%) |  |
|  |  |  |  |  |
| **Hypochloremia** |  |  |  | 1.00 |
| No | 7 (87.5%) | 8 (100.0%) | 15 (93.8%) |  |
| Yes | 1 (12.5%) | 0 (0.0%) | 1 (6.3%) |  |
|  |  |  |  |  |
| **Hypokalemia** |  |  |  | 1.00 |
| No | 6 (75.0%) | 6 (75.0%) | 12 (75.0%) |  |
| Yes | 2 (25.0%) | 2 (25.0%) | 4 (25.0%) |  |
|  |  |  |  |  |
| **Hyponatremia** |  |  |  | 1.00 |
| No | 7 (87.5%) | 8 (100.0%) | 15 (93.8%) |  |
| Yes | 1 (12.5%) | 0 (0.0%) | 1 (6.3%) |  |
|  |  |  |  |  |
| **Hypophosphatemia** |  |  |  | 1.00 |
| No | 7 (87.5%) | 8 (100.0%) | 15 (93.8%) |  |
| Yes | 1 (12.5%) | 0 (0.0%) | 1 (6.3%) |  |
|  |  |  |  |  |
| **Hypoxemia** |  |  |  | 1.00 |
| No | 8 (100.0%) | 7 (87.5%) | 15 (93.8%) |  |
| Yes | 0 (0.0%) | 1 (12.5%) | 1 (6.3%) |  |
|  |  |  |  |  |
| **Ischemic stroke** |  |  |  | 0.47 |
| No | 6 (75.0%) | 8 (100.0%) | 14 (87.5%) |  |
| Yes | 2 (25.0%) | 0 (0.0%) | 2 (12.5%) |  |
|  |  |  |  |  |
| **Metabolic alkalosis** |  |  |  | 1.00 |
| No | 8 (100.0%) | 7 (87.5%) | 15 (93.8%) |  |
| Yes | 0 (0.0%) | 1 (12.5%) | 1 (6.3%) |  |
|  |  |  |  |  |
| **Occulussive thrombus in vein** |  |  |  | 1.00 |
| No | 8 (100.0%) | 7 (87.5%) | 15 (93.8%) |  |
| Yes | 0 (0.0%) | 1 (12.5%) | 1 (6.3%) |  |
|  |  |  |  |  |
| **Somnalence** |  |  |  | 1.00 |
| No | 7 (87.5%) | 8 (100.0%) | 15 (93.8%) |  |
| Yes | 1 (12.5%) | 0 (0.0%) | 1 (6.3%) |  |
|  |  |  |  |  |
| **Tachycardia** |  |  |  | 1.00 |
| No | 7 (87.5%) | 8 (100.0%) | 15 (93.8%) |  |
| Yes | 1 (12.5%) | 0 (0.0%) | 1 (6.3%) |  |
|  |  |  |  |  |
| **Terson's Syndrome** |  |  |  | 1.00 |
| No | 7 (87.5%) | 8 (100.0%) | 15 (93.8%) |  |
| Yes | 1 (12.5%) | 0 (0.0%) | 1 (6.3%) |  |
|  |  |  |  |  |
| **Urinary Tract Infection** |  |  |  | 0.47 |
| No | 8 (100.0%) | 6 (75.0%) | 14 (87.5%) |  |
| Yes | 0 (0.0%) | 2 (25.0%) | 2 (12.5%) |  |
|  |  |  |  |  |
| **Vasospasm** |  |  |  | 1.00 |
| No | 6 (75.0%) | 7 (87.5%) | 13 (81.3%) |  |
| Yes | 2 (25.0%) | 1 (12.5%) | 3 (18.8%) |  |
|  |  |  |  |  |
| **Visual hallucinations** |  |  |  | 1.00 |
| No | 8 (100.0%) | 7 (87.5%) | 15 (93.8%) |  |
| Yes | 0 (0.0%) | 1 (12.5%) | 1 (6.3%) |  |

**Supplemental Figure Legends**

**Supplemental Figure 1.** Standard of Care Pain Management Regimen with NPS Titration algorithm. *Unless serum creatinine >1.3, glomerular filtration rate <30, concern for gastrointestinal bleed risk, or history of bleeding peptic ulcer. Abbreviations: BID indicates twice a day; hrs, hours; IV, intravenous; NPS, numeric pain score; PRN, as needed; q, every.

**Supplemental Figure 2.** Study Timeline of Events. hr indicates hour; NPS, numeric pain score.

**Supplemental Figure 3.** CONSORT Flow Diagram.
